# Wide Residual Relation Network-Based Intelligent Fault Diagnosis of Rotating Machines with Small Samples

**DOI:** 10.3390/s22114161

**Published:** 2022-05-30

**Authors:** Zuoyi Chen, Yuanhang Wang, Jun Wu, Chao Deng, Weixiong Jiang

**Affiliations:** 1 School of Mechanical Science and Engineering, Huazhong University of Science and Technology, Wuhan 430074, China; zuoyihust@163.com (Z.C.); dengchao@hust.edu.cn (C.D.); 2 China Electronic Product Reliability and Environmental Testing Research Institute, Guangzhou 510610, China; wangyuanhang@ceprei.com; 3 School of Naval Architecture and Ocean Engineering, Huazhong University of Science and Technology, Wuhan 430074, China; jiangweixiong@hust.edu.cn

**Keywords:** rotating machines, fault diagnosis, few-shot learning, wide residual network, relational network

## Abstract

Many existing fault diagnosis methods based on deep learning (DL) require numerous fault samples to train the diagnosis model. However, in industrial applications, rotating machines (RMs) operate in normal states for most of their service life with fault events being rare and thus failure samples are very limited. To solve the problem above, a novel wide residual relation network (WRRN) is proposed for intelligent fault diagnosis of the RMs. Specifically, the WRRN is trained by performing a series of learning tasks in RMs with sufficient samples to obtain knowledge about how to diagnose, and then it is directly transferred to realize fault task of the RM with small samples. In this method, a wide residual network-based feature extraction module is used to generate representative fault features from input samples, and a relation module is designed to calculate the relation score between the sample pairs so as to determine their categories. Extensive experiments are conducted on two RMs to validate the WRRN method. The results demonstrate that the WRRN can accurately identify the fault types of the RMs with only small samples or even one sample. The WRRN significantly outperforms the existing popular methods in diagnostic performance.

## 1. Introduction

As a multi-disciplinary research field, rotating machine (RM) fault diagnosis has been explored by studies from mechanical engineering, machine learning, artificial intelligence, fault tolerance schemes, and so on [1,2,3,4,5]. Many machine-learning (ML) methods have been employed and modified for fault diagnosis. The ML-based fault diagnosis methods generally include feature extraction and classifier [6,7,8]. The widely used feature extraction methods include empirical mode decomposition [9], Fourier transform [10], continuous wavelet transform (CWT) [11], and so on. The classifier-based ML contain support vector machine [12], Bayesian [13], ensemble learning [14], and so on. However, these ML-based fault diagnosis methods require manually extracted features, which cannot provide an end-to-end diagnosis.

In recent decades, intelligent fault diagnosis methods based on deep learning (DL) have become widely applied. Many DL methods for fault diagnosis in industrial machines has been received attention due to their ability to automatically extract fault features from monitoring signals and deliver reliable diagnostic results [15,16,17]. For instance, Li et al. [18] incorporated Bayesian Gaussian mixture and convolutional neural network (CNN) to perform bearing fault diagnosis. A bearing dataset and a gearbox dataset are used to test the efficiency of the proposed method. Chen et al. [19] combined continuous wavelet transform and local binary CNN to provide end–end fault diagnosis of RMs. Two experimental studies are conducted to verify the stability and reliability of the proposed method, including bearing fault diagnosis and gearbox compound fault diagnosis. Zhao et al. [20] developed a deep network based on a residual shrinkage network. A soft threshold is inserted into the network to eliminate unimportant features, resulting in improved feature learning ability from highly noised signals and high fault diagnostic accuracy. Most existing DNN-based fault diagnosis methods aim to improve diagnostic accuracy given enough fault samples, while restricted fault samples are rarely considered [21,22,23]. However, industrial applications, RMs operate under the normal state in most of their service life, whereas failure events seldom happen. Thus, it is difficult to collect sufficient fault samples to meet the training purposes of DL models.

Transfer learning (TL) can transfer the diagnostic knowledge learned from source domain to apply it to a related but new target domain for fault diagnosis [24,25,26]. Many researchers have found that the transfer diagnostic model is formed on RM operated in lab environment (RMLE) and then transferred to specific machines with small fault samples for fault diagnosis. Yang et al. [27] developed a feature-based transfer neural network (FTNN) for bearing fault diagnosis. The FTNN can learn diagnostic knowledge from other machines to diagnose the health of the machine. Shao et al. [28] used CWT to transform time-frequency maps from raw vibration signals. Then, the TL model based on DL has been built and high diagnostic accuracy obtained on three datasets including gearboxes, motors, and bearings. Guo et al. [29] proposed a transfer relation network and employed multikernel maximum mean discrepancy to improve the transfer performance. The effectiveness of the method is verified by four datasets, including three lab datasets and one practical dataset. Other transfer learning-based tasks were also investigated, such as motor fault diagnosis [30] and tool remaining useful life prediction [31]. Those TL-based fault diagnosis methods required a certain correlation between the source domain and the target domain, and the data from the target domain involved in training. However, it is hard to find an appropriate dataset as source domain. Since failure events are uncommon in real-world industrial scenarios, it is difficult to ensure that the target machine dataset has a certain number of fault samples.

For the problem of small data, few-shot learning methods have been proven to be an effective solution by many researchers [32,33]. The few learnings provide much practical value and have recently received a lot of attention from researchers in the field of computer vision. The few-shot methods are able to learn classifiers in source domains with enough labeled data and then perform a classification task on target domains with little labeled data of each class. Li et al. [34] developed a hierarchical Bayesian model to learn visual concepts with just one example. Gregory et al. [35] developed a Siamese network for one shot learning. This network used a similarity algorithm to measure the similarity between samples. Sung et al. [36] designed a relation network to calculate the relation score between the sample pairs to determine their types.

Inspired by the above-mentioned methods for few-shot learning methods, a novel wide residual relation network (WRRN) is proposed in this paper for solving the few sample problems in intelligent fault diagnosis of RMs. The method mainly includes a feature extraction module and a relation module. The wide residual network-based feature extraction module is used to generate representative fault features from input samples. The relation module calculates the relation score between the sample pairs to determine their types. The main contributions of this paper are summarized below.

1. A WRRN method is first proposed to exploit the fault knowledge learned from the lab machine for fault diagnosis in several real-case machines with small fault data, whereas only lab machine datasets are used for training.
2. The built wide residual network can generate more representative fault features from input samples compared to traditional CNN methods.
3. The relation module can reveal the similarity relations between the sample pairs to determine their categories, which can improve diagnostic performance.

The remainder of the paper is organized as follows. Section 2 describes problem definition, the proposed WRRN method and the optimization objective of the WRRN. Section 3 presented a fault diagnosis procedure based on WRRN. In Section 4 discusses the experimental results. Finally, Section 5 summarizes the proposed method.

## 2. Proposed Method

### 2.1. Problem Formulation

The dataset for the WRRN method mainly includes training dataset $D_{train}$ and the test dataset $D_{test}$, where the dataset $D_{train}$ is a relatively large labeled dataset from RMLE and the dataset $D_{test}$ is only a small dataset from RM operated in real-world environments (RMRE). The purpose of the WRRN is to can utilize the transferable diagnostic model trained on the dataset $D_{train}$ to the test datasets $D_{test}$ for fault diagnosis. Both training dataset and the test datasets include support set S and query set ℚ. The $D_{train}$ and the $D_{test}$ can be redefined as $D_{train}=\left\{S_{train},{\mathbb{Q}}_{train}\right\}$ and $D_{test}=\left\{S_{test},{\mathbb{Q}}_{test}\right\},$ respectively, where the S represents labeled dataset, and the ℚ represents unlabeled dataset. The support set $S={\left\{\left(x_{i},y_{i}\right)\right\}}_{i=1}^{m}$ consists of C health conditions, each health conditions with K labeled samples. For each episode, the total number of S samples have m=K×C. The few-shot learning method is to diagnose the health conditions of the ℚ based on the S. This setting can be called C-way K-shot diagnosis.

For each training episode, a certain number of samples are randomly selected from the $D_{train}$ to construct a C-way K-shot setting as follows: $T=S_{train}\cup {\mathbb{Q}}_{train}$ where $S_{train}={\left\{\left(x_{i},y_{i}\right)\right\}}_{i=1}^{m}$ and $\;{\mathbb{Q}}_{train}={\left\{\left(x_{j},y_{j}\right)\right\}}_{j=1}^{n}$. $\left|T\right|=m+n$ is the total number of the samples in the task T. In the training process, the WRRN model $F\left(S,\mathbb{Q};\vartheta \right)$ is learned on the $S_{train}$ labeled to minimize the predictions loss of ${\mathbb{Q}}_{train}$.

(1) $$F\left(S,\mathbb{Q};\vartheta \right):\left(S_{train},{\mathbb{Q}}_{train}\right)\to C_{train}^{\mathbb{Q}}$$

where $C_{train}^{\mathbb{Q}}$ represents the health conditions of the ${\mathbb{Q}}_{train}$. θ is indicated as

(2) $$\vartheta =\underset{\theta }{\mathrm{argmin}}\sum_{D_{train}}S\left(S_{train},{\mathbb{Q}}_{train}\right)$$

where $S\left(\cdot \right)$ represents the similarity between the $S_{train}$ and the ${\mathbb{Q}}_{train}$.

For test process, the WRRN model ϑ is transferred to diagnose the test dataset $D_{test}=\left\{S_{test},{\mathbb{Q}}_{test}\right\}$, where the $S_{test}$ represents small-labeled samples, and the ${\mathbb{Q}}_{test}$ needs to be diagnosed.

(3) $$F\left(S,\mathbb{Q};\vartheta \right):\left(S_{test},{\mathbb{Q}}_{test}\right)\to C_{test}^{\mathbb{Q}}$$

In this paper, the assumptions are given as:

1. The different RMs have the same machine health states.
2. The training dataset comes from a RMLE. The test dataset is from a RMRE, which is not required to be involved in the training process.

### 2.2. Wide Residual Relation Network

**Figure 1:**
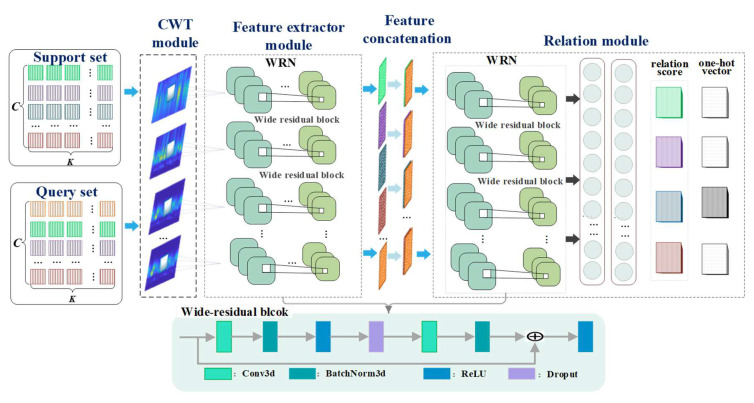
Structure illustration of the proposed TRPGN model.

As illustrated in Figure 1, the WRRN consists of a CWT module $W_{\delta }$, feature extractor module $F_{\varphi }$ and relation module $R_{\theta }$. The CWT module $W_{\delta }$ is adopted to convert time-frequency maps from raw signals. The feature extractor $F_{\varphi }$ can mine time-frequency maps to generate representative fault features. Then the features are fed into the relation module $R_{\theta }$. The relation module $R_{\theta }$ calculates the similarity relations between the sample pairs to determine their categories. The WRRN is described in detail below.

**CWT module:** The CWT can reveal fault information at low-frequency and high-frequency information, preserving the effective signal features. In the CWT module, wavelet time-frequency maps are formed by calculating the inner product of the time-domain data $x\left(t\right)$ and the wavelet basis function $\varphi_{u,s}\left(t\right)$, which are expressed as

(4) $$W_{\delta }\left(u,s\right)=\frac{1}{\sqrt{s}}\int_{-\infty }^{-\infty }x\left(t\right)\psi \left(\frac{t-u}{s}\right)dt$$

where s is the scale factor of CWT, and u is the time shift factor. Then, the support set $S={\left\{\left(x_{i},y_{i}\right)\right\}}_{i=1}^{m}$ and the query set $\mathbb{Q}={\left\{\left(x_{j},y_{j}\right)\right\}}_{j=1}^{n}\;$ are converted to wavelet time-frequency maps by CWT and fed to the feature extractor module.

**Feature extractor:** The feature extractor $F_{\varphi }$ is adopted to mine useful information and extract high-level features from the wavelet time-frequency maps. The specific structural parameters of the feature extractor $F_{\varphi }$ are shown in Table 1. The feature extractor $F_{\varphi }$ adoptes a wide residual network (WRN), which consists of four wide residual blocks and one pooling block. The operation of wide residual block is formulated as

(5) $$W_{l}\left(x_{i}^{l}\right)=F\left(c^{l}x_{i}^{l}+b^{l}\right)$$

where $W_{l}\left(x_{i}^{l}\right)$ is convolutional operation. $c^{l}$ and $b^{l}$ represent the convolutional kernel and bias at layer l, respectively. The operation of pooling block is defined as

(6) $$P_{l}\left(x_{i}^{l}\right)=Avgx_{a,b}\left(x_{i}^{l}\right)$$

where  a and b denote the length and width of the pool window, respectively. For inputs of the support set $S={\left\{\left(x_{i},y_{i}\right)\right\}}_{i=1}^{m}$ and the query set $\mathbb{Q}={\left\{\left(x_{j},y_{j}\right)\right\}}_{j=1}^{n}$, the corresponding outputs of the feature extractor $F_{\varphi }$, are described as high-level features $f_{S}={\left\{f_{i}\right\}}_{i=1}^{m}$ and level features $f_{\mathbb{Q}}={\left\{f_{j}\right\}}_{j=1}^{n}$, respectively.

To explicitly indicate the feature relations between support set and query set, support–query pairs are constructed. The support–query pairs are represented as

(7) $$G_{m}\left(f_{S},f_{\mathbb{Q}}\right)=\left[f_{i},f_{j}\right]$$

where $f_{i}\in f_{S}={\left\{f_{i}\right\}}_{i=1}^{m}$, $g_{j}\in f_{\mathbb{Q}}={\left\{f_{j}\right\}}_{j=1}^{n}$. $\left[\cdot ,\cdot \right]$ is the concatenation operation.

**Relation module:** The relation module $R_{\theta }$ is composed of WRN and two fully connected layers. The WRN is composed of two wide residual blocks, which can mine relational features of support–query pairs. The specific structural parameters of the relation module $R_{\theta }$ are shown in Table 1. The corresponding operation is shown in Equation (5). For inputs of the support–query pairs $G_{m}$, the corresponding outputs of the WRN are described as relational features $f_{G}={\left\{f_{i}\right\}}_{i=1}^{m}$. The relational features $f_{G}={\left\{f_{i}\right\}}_{i=1}^{m}$ are fed into connected layers to the relation score about support–query pairs. The sizes of two fully connected blocks is 1×8 and 1×1, respectively. The output of the fully connected blocks in l is expressed as

(8) $$G_{l}\left(x_{i}^{l}\right)=f\left({ο}^{l}x_{i}^{l}+\rho^{l}\right)$$

where ${ο}^{l}$ and $\rho^{l}$ represent the weight and bias of fully connected blocks at layer l. The relation module $R_{\theta }$ can calculate the relation score about feature map pairs $G_{m}$ to preform relationship learning. The relation score $r_{i,j}$ is a scalar between 0 and 1 and represents the similarity between the support set and the query set. This means that the higher relation score belongs to the same category, while the lower relation score belongs to a different category. Thus, the output of the relation module $R_{\theta }$ is defined as

(9) $$r_{i,j}=R_{\theta }(S\left(G_{n}\left(f_{S},f_{\mathbb{Q}}\right)\right),\;i=1,2,\ldots ,C$$

### 2.3. Optimization Objective of the WRRN

Considering that the WRRN is a similarity score regression task, mean square error (MSE) is adopted to calculate loss function of the WRRN. The loss function is formulated as

(10) $$L_{MSE}=\sum_{i=1}^{m}\sum_{j=1}^{n}{\left(r_{i,j}-1\cdot \left(y_{i}==y_{j}\right)\right)}^{2}$$

If $y_{i}$ and $y_{j}$ are in the same category, the label is 1; otherwise, the label is 0.

Suppose $\theta_{F_{\varphi }}$ and $\theta_{R_{\theta }}$ are the parameters of the feature extractor module $F_{\varphi }$ and the relation module $R_{\theta }$, respectively. The (8) is rewritten as

(11) $$\theta_{F_{\varphi }}^{*},\theta_{R_{\theta }}^{*}\leftarrow \underset{\theta_{F_{\varphi }},\theta_{R_{\theta },}}{\mathrm{argmin}}L_{MSE}\left(\theta_{F_{\varphi }},\theta_{R_{\theta }}\right)$$

where $\theta_{F_{\varphi }}^{*}$ and $\theta_{R_{\theta }}^{*}$ are optimal parameters. The training pipeline of the WRRN in an epoch is described in Algorithm 1.
**Algorithm 1**. Mini-batch training algorithm for the WRRN. b and epochs denote the batch size and the number of iterations**Input:** support set $S_{train}={\left\{\left(x_{i},y_{i}\right)\right\}}_{i=1}^{m}$; query set ${\mathbb{Q}}_{train}={\left\{\left(x_{j},y_{j}\right)\right\}}_{j=1}^{n}$; Feature extractor module $F_{\varphi }$; relation module $R_{\theta }$. **for** i=1 to epochs do  Randomly sample K support set $S_{train}={\left\{\left(x_{i},y_{i}\right)\right\}}_{i=1}^{m}$ and N query set ${\mathbb{Q}}_{train}={\left\{\left(x_{j},y_{j}\right)\right\}}_{j=1}^{n}$ from each category  of $D_{train}$ to construct b;  Forward update $L_{MSE}\left(\theta_{F_{\varphi }},\theta_{R_{\theta }}\right)$  Backward update $F_{\varphi }$ and $R_{\theta }$**;****end for****return** $F_{\varphi }$ and $R_{\theta }$ for the classification of test datasets

## 3. Fault Diagnosis Procedure Based on WRRN

**Figure 2:**
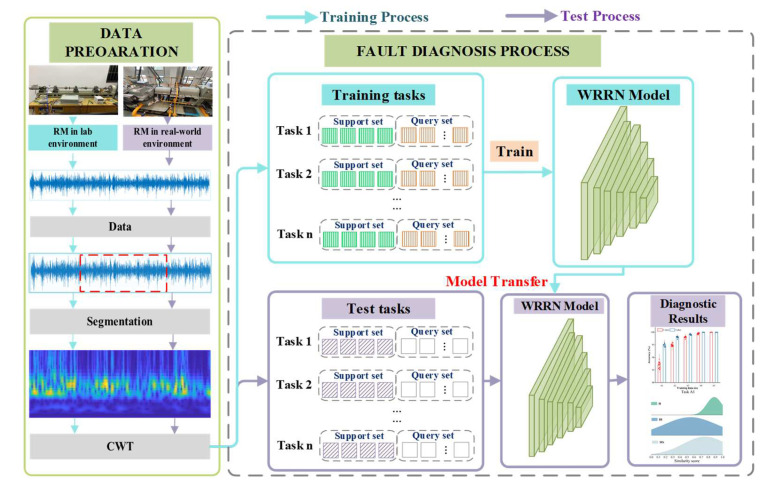
Fault diagnosis pipeline based on the WRRN method.

As shown in Figure 2, the WRRN includes the training process and test process. In the training process, the WRRN model is trained on the dataset from the RMLE. In the test process, the trained WRRN model is transferred to diagnose the health conditions of the RMLE. These two processes are described below.

In the training process, fault simulation experiments are conducted on RMLE to generate fault data of different fault types. The simulated fault types by the RMLM need to include all fault types occurring in the RMRM, but do not require the same type of machine as the RMRM. The RMLE dataset is used as the training dataset $D_{train}$. Next, the dataset $D_{train}$ are converted by CWT into wavelet time–frequency maps with a size of 28 × 28. Correspondingly, the support set $S_{train}={\left\{\left(x_{i},y_{i}\right)\right\}}_{i=1}^{m}$ and the query set ${\mathbb{Q}}_{train}={\left\{\left(x_{j},y_{j}\right)\right\}}_{j=1}^{n}$ are constructed. The built WRRN model is trained on the $S_{train}$ and ${\mathbb{Q}}_{train}$. The WRRN model is trained in such way that the final loss $L_{MSE}$ is minimized, and the training process is completed. This trained WRRN model is directly used to diagnose the health conditions of the RMLEs.

In the test process, the test dataset $D_{test}$ is collected from the RMRE, where the RMRE has a very small quantity of labeled samples. The test datasets $D_{test}$ are converted by CWT into wavelet time–frequency maps with a size of 28 × 28. The support set $S_{test}={\left\{\left(x_{i},y_{i}\right)\right\}}_{i=1}^{m=K\times C}$ and the query set ${\mathbb{Q}}_{test}={\left\{\left(x_{j}\right)\right\}}_{j=1}^{n}$ are constructed from the test dataset $D_{test}$. The $S_{test}={\left\{\left(x_{i},y_{i}\right)\right\}}_{i=1}^{m}$ represents the small-labeled data from the RMRE. The ${\mathbb{Q}}_{test}={\left\{\left(x_{j}\right)\right\}}_{j=1}^{n}$ represents the diagnosed data. Both sets are fed into the trained WRRN model. The WRRN model can calculate the similarity score between the ${\mathbb{Q}}_{test}$ and the labeled $S_{test}$ to figure out the health conditions of the ${\mathbb{Q}}_{test}$.

## 4. Experimental Studies

### 4.1. Experimental Setup and Dataset Description

The WRRN method is validated by RMs from two different fields, including a shafting machine and a steam turbine.

**Figure 3:**
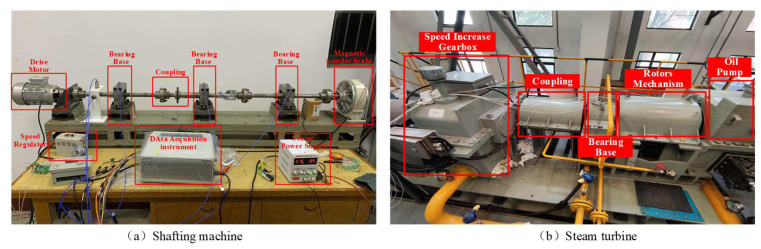
Test bench for RMs: (**a**) the shafting machine operated in lab environment; (**b**) the steam turbine operated in real-world environments.

The shafting machine is a self-built testbed to obtain large amounts of labeled data by simulating failure experiments. The shafting machine consists of three intermediate bearings, a magnetic powder brakes, a flange, and a drive motor, as shown in Figure 3a. The shafting machine dataset has three machine health conditions: misalignment (MS), imbalance (IB), and normal (N). The shafting machine operates under five operating conditions controlled by the speed of the shaft. Variation signals are collected for 2 min at 2000 Hz. A total of 1000 samples are obtained each with 1024 data points for each health condition. Table 2 shows a detailed description of the dataset.

As shown in Figure 3b, the steam turbine consists of a speed increasing gearbox, a rotor mechanism, a coupling, a bearing based, an electric motor, and an oil pump. The rotation seep of motor is 6680 r/min, and the flow rate of the oil circuit system is 1300 L/min. The steam turbine has three health conditions, including N, IB, and MS. Variation signals are collected at 20 kHz. A total of 1000 samples each with 1024 data points is collected for each health condition. Table 2 shows a detailed description of the steam turbine dataset.

Considering that the shafting machine dataset is recorded from the RMLE, and the steam turbine dataset is from the RMRE, the transfer experiments of shafting machine to steam turbine are carried out. The shafting machine dataset is collected under five operating conditions. Thus, Table 3 lists five transfer tasks: A1, A2, A3, A4, and A5. For instance, the task A1 represents the shafting machine data from the L1 operating condition as the training dataset and the steam turbine dataset as the test dataset.

### 4.2. Results and Discussion

To explore the impact of the WRN as feature extractors on the diagnostic performance of the WRRN, a comparative experiment with the CNNRN method using the CNN as feature extractors is carried out. The experiment settings of 1-shot, 3-shot, 5-shot, 8-shot, and 10-shot are carried out to investigate the impact of the WR as feature extractors on the diagnostic performance of the WRRN method. For instance, 1-shot represents that one sample from each health state in the shafting machine is taken as the support set $S_{train}$ for the training process. For the test process, the 1-shot indicates that only one sample from each health state in the steam turbine is labeled and taken as the support set $S_{test}$ for test process. Taking task A1 as an example, the impact of the fault sample size on the diagnostic performance of the WRRN method is explored. For each experiment setting, the training strategy of the WRRN method follows the usual zero-shot learning way by episode-based training. These two methods adopt Adam with an initial learning rate $10^{-3}$ and half annealing every 1000 sets for end-to-end training.

**Figure 4:**
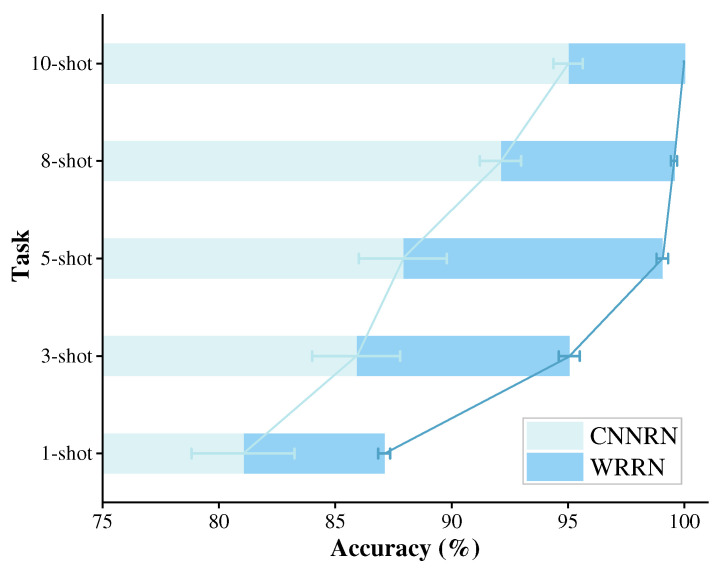
Diagnostic performance comparison on methods CNNRN and WRRN.

Figure 4 displays the diagnostic mean accuracies and standard deviations (Std) of the WRRN method and the CNNRN method, where both methods are conducted in ten trials. The results reveal that the diagnostic accuracies of the WRRN method are significantly higher than those of the CNNRN method by at least 5% in the five diagnostic tasks. From the Std perspective, it is also seen that the diagnostic performance of the WRRN method is significantly more stable than that of the CNNRN method. This can prove that the WRN has superior feature extraction capability, which enables the relation module to better discriminate the similarity relations between sample pairs, so as to improve the diagnostic performance of the WRRN. Furthermore, as the fault sample size from the steam turbine increases, the diagnostic accuracy improves. The WRRN method achieves almost 100% diagnostic accuracy, and the lowest Std in task 10-shot. The conclusion from the results is that increasing the fault sample size significantly improves the diagnostic performance of the WRRN method.

**Figure 5:**
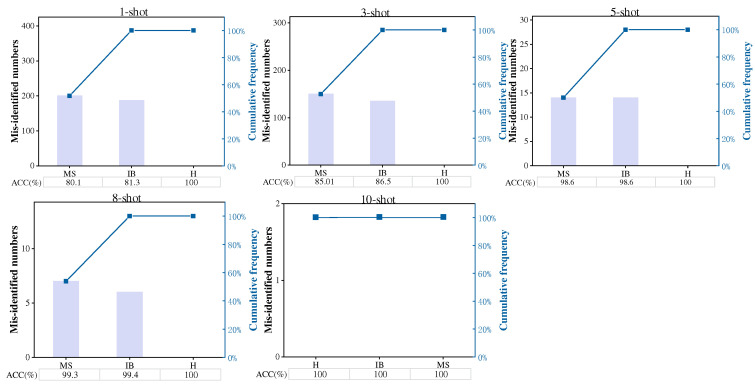
Diagnostic performance of WRRN method for five settings under task A1.

Figure 5 shows the Pareto charts of diagnostic performance of the WRRN method for five settings under task A1. Each plot in Figure 5 represents the number of misidentified testing samples in all health conditions from largest to smallest, and the cumulative frequency of misidentified samples. The results also show that most of the fault samples with incorrect diagnoses are related to the inner race fault samples, while the misdiagnosis rate between the health samples and the outer race fault samples is much smaller. This implies that the WRRN method can accurately diagnose the health state and fault state, which is a critical need in real-world engineering applications. These results demonstrate that the WRRN trained on the shafting machine can be directly transferred to the steam turbine for fault diagnosis and achieve superior diagnosis performance.

**Figure 6:**
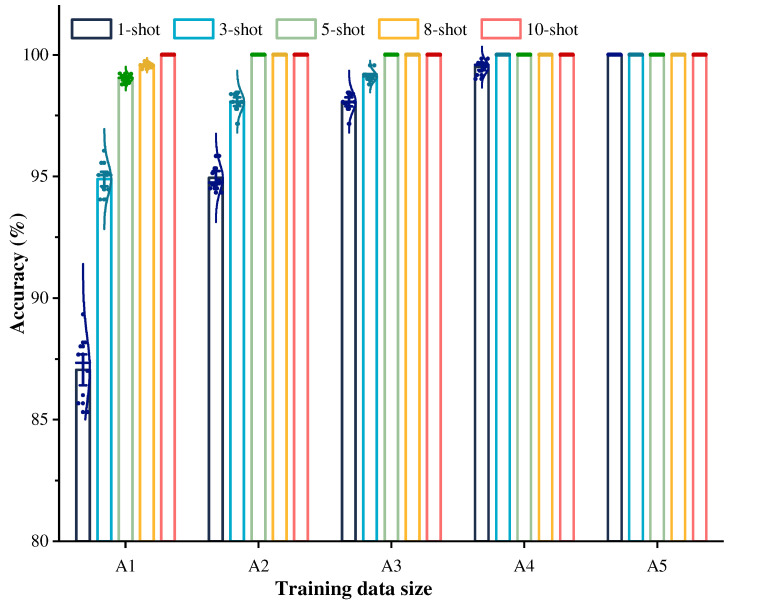
Diagnostic performance of the WRRN method under different training dataset size for the different setting.

It can be seen from Figure 4 that the diagnostic accuracy of the WRRN method is relatively low on the 1-shot and 3-shot settings, especially the diagnostic accuracy of the WRRN method on the 1-shot is only 87.1%. Therefore, it is explored to improve the diagnostic performance of the WRRN method by increasing the training data size when the fault samples of the steam turbine are relatively small. Figure 6 displays the diagnostic mean accuracy and standard deviations (Std) for the different settings. It can be shown from Figure 6 that as the training dataset size increases, the diagnostic mean accuracy increases, and the Std decreases accordingly. The WRRN method achieves almost 100% diagnostic accuracy and the lowest Std in task A5. It can be concluded that the diagnostic performance of the WRRN method for the steam turbine can be improved by increasing the training data size when there is only one sample of each fault type from the steam turbine. In addition, it can be found that the diagnostic accuracy of the WRRN method under 10-shot setting is as high as 100% when the task A1. It can also be concluded that when there is a certain amount of each fault type from the steam turbine, the WRRN method can also achieve effective diagnostic performance.

**Figure 7:**
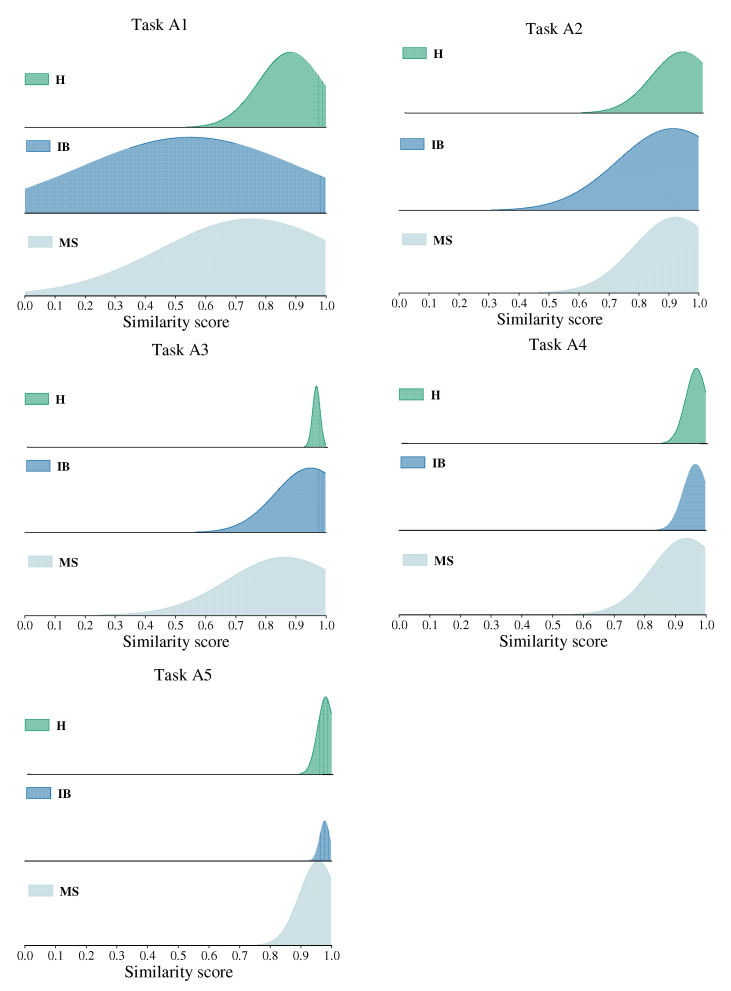
Distribution of similar scores between each health state.

Figure 7 displays the distribution of similar scores between each health state. It can be concluded from the Figure 7 that the similarity scores of each category increase gradually with the increase of the training data size. The similarity scores for each category in task A5 are concentrated at 0.9. This verifies that the diagnostic accuracy of the WRRN method is as high as 100% in task A5. Thus, the diagnostic performance of the WRRN method can be improved by increasing the amount of training data from the shafting machine when fault samples from the steam turbine are severely insufficient.

Table 4 displays the classification time for each sample under the different setting. All experiment methods are performed on a NVIDIA GeForce GTX 1660, a computer (Intel Core (TM) 3.6 GHz processor with 8 GB of RAM), and a windows version of the PyTorch platform. As can be seen from Table 4, as the number of supporting samples increases, the classification time for each sample increases accordingly. If the diagnosis task requires fast classification time, the number of supporting samples can be reduced, and the training dataset can be increased to ensure effective fault diagnosis performance.

### 4.3. Comparative Analysis

To show the superiority of the WRRN method even more clearly, several advanced methods for comparison, including DL methods, TL methods, and few-shot learning methods, are used to demonstrate the superiority of the WRRN method. The DL adopts the WRN as feature extractor like the WRRN method. For the WRRN method, the training dataset includes a shafting machine dataset and a small amount of fault data from the steam turbine. For TL methods, VGG-11 [37] and Resnet-18 [38] are used as backbone networks for knowledge transfer. The two networks are pre-trained on the data from the shafting machine, and then fine-tuned on small data from the RM operating real-world experiment. For the few-shot learning methods, the matching network [39] is employed in the comparative experiments, using the same feature extractors as the WRRN method. For a fair comparison, the C-way K-shot setting is used for all the above comparing methods.

**Figure 8:**
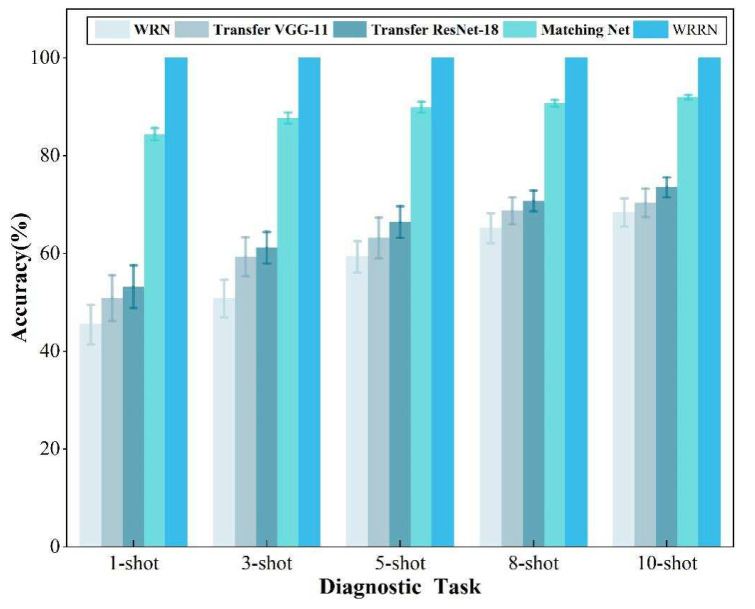
Accuracy comparison results of different methods on task A5.

The task A5 is chosen as a comparison experiment because the fault data from the shafting machine could be simulated. The Figure 8 shows the fault diagnosis performance of different methods in the steam turbine with different fault sample sizes. Similarly, each method is tested in ten trials, and the diagnostic accuracies and standard errors are obtained for six methods. It can be found from the Figure 8 that the diagnostic accuracy of all methods increases with the increase of the fault samples from the steam turbine. The diagnostic performance of these few-shot learning approaches is significantly better than that of these DL and TL methods. In terms of diagnostic performance, the WRRN method beats all other methods, with diagnostic accuracy approaching 100% in a variety of fault sample sizes. This is because the wide residual network can generate more representative fault features from input samples. Then, RM can reveal the similarity relations between the features pairs to determine their categories, which can improve diagnostic performance. These findings further show that the WRRN method can accurately diagnose health conditions of the steam turbine when just few fault samples or even only one fault sample is provided.

## 5. Conclusions

In this paper, a novel WRRN is proposed to diagnose the health conditions of RM with insufficient fault data. Specifically, the WRRN is trained by performing a series of learning tasks in RMs with sufficient samples to obtain knowledge about how to diagnose, and then, it is directly transferred to diagnose the RM with small samples. The method mainly includes the feature extraction module and the relation module. The wide residual network-based feature extraction module is used to generate representative fault features from input samples. The relation module calculates the relation score between the sample pairs so as to determine their health states. Extensive experiments are conducted on two RMs to validate the WRRN method. The results show that the WRRN model trained on a RMLE can properly diagnose the health conditions of RMRE with just a few fault samples, where the RMRE and the RMLE can come from different machine domains. Furthermore, the impact of RMRE fault sample size and training dataset size on diagnostic performance have been investigated. The results show that increasing the RMRM fault sample size or the RMLE training dataset size can improve the diagnostic performance of the WRRN significantly. Finally, the comparative experiments demonstrate that the WRRN outperforms state-of-the-art methods for fault diagnosis in RMs with very limited fault data circumstances.

## Figures and Tables

**Table 1:** Architecture of feature extractor and relation module.

Module	Group Name	Block Type = B (3,3)
Feature extractor $F_{\varphi }$	Conv_1	$\left[3\times 3,\;16\right]$
	Conv_2	$\left[\begin{array}{cc} 3\times 3, & 16\times 2\\ 3\times 3, & 16\times 2 \end{array}\right]\times N$
	Conv_3	$\left[\begin{array}{cc} 3\times 3, & 16\times 3\\ 3\times 3, & 16\times 3 \end{array}\right]\times N$
	Conv_4	$\left[\begin{array}{cc} 3\times 3, & 16\times 4\\ 3\times 3, & 16\times 4 \end{array}\right]\times N$
	Avg-pool	$\left[5\times 5\right]$
Relation module $R_{\theta }$	Conv_1	$\left[\begin{array}{cc} 3\times 3, & 16\times 4\\ 3\times 3, & 16\times 4 \end{array}\right]\times N$
	Conv_2	$\left[\begin{array}{cc} 3\times 3, & 16\times 4\\ 3\times 3, & 16\times 4 \end{array}\right]\times N$
	Avg-pool	$\left[1\times 1\right]$
	FC 1	8
	FC 2	1

**Table 2:** Introduction to datasets of lab machines and real-case machines.

Datasets	Health State	Operating Conditions	Number of Samples
Shafting machine	N I B MS	L1–200 r/min	3 × 1000
		L2–250 r/min	3 × 1000
		L3–300 r/min	3 × 1000
		L4–350 r/min	3 × 1000
		L5–400 r/min	3 × 1000
Steam turbine	N IB MS	6680 r/min 1300 L/min	3 × 1000

**Table 3:** Description of fault diagnosis task.

Task	Training Dataset from Shafting Machine	Testing Dataset
A1	L1	Steam turbine
A2	L1, L2	Steam turbine
A3	L1, L2, L3	Steam turbine
A4	L1, L2, L3, L4	Steam turbine
A5	L1, L2, L3, L4, L5	Steam turbine

**Table 4:** Classification time for each sample under the different setting.

Task	Classification Time (ms)
1-shot	4.1
3-shot	19.5
5-shot	44.5
8-shot	64.25
10-shot	90.25

## Data Availability

Not applicable.
